# Blood MALT1 expression could help predict treatment outcomes in psoriasis patients, especially in those receiving biologics

**DOI:** 10.1002/iid3.1235

**Published:** 2024-04-05

**Authors:** Qiaoli Liu, Yanfeng Zhang, Bing Xu, Xiaobo Jin, Tao Yang, Leiqiang Fan

**Affiliations:** ^1^ Department of Emergency Chengde Central Hospital Chengde China; ^2^ Department of Dermatology Chengde Central Hospital Chengde China; ^3^ Department of Orthopaedics Chengde Central Hospital Chengde China; ^4^ Department of Clinical Laboratory Chengde Central Hospital Chengde China

**Keywords:** biologic therapy, disease activity, mucosa‐associated lymphoid tissue 1, psoriasis, treatment response

## Abstract

**Introduction:**

Mucosa‐associated lymphoid tissue 1 (MALT1) modulates T helper cell differentiation, pro‐inflammatory cytokine production, and epidermal hyperplasia to participate in the pathology of psoriasis. This study aimed to explore the correlation of blood MALT1 with treatment outcomes in psoriasis patients.

**Methods:**

MALT1 was detected in peripheral blood mononuclear cells by reverse transcription‐quantitative polymerase chain reaction in 210 psoriasis patients before starting or converting to a new therapy, 50 disease controls, and 50 healthy controls. The psoriasis area severity index (PASI) score was evaluated at month (M)1, M3, and M6 in psoriasis patients.

**Results:**

MALT1 was increased in psoriasis patients versus disease controls and healthy controls (both *p* < .001); and positively related to body mass index (*p* = .019) and PASI score (*p* < .001) in psoriasis patients. PASI75 rate at M1, M3, and M6 was 22.9%, 46.2%, and 71.0%, respectively; while PASI90 rate at M1, M3, and M6 was 3.8%, 29.0%, and 50.5%, respectively, in psoriasis patients. PASI75/90 rates at M1, M3, and M6 were increased in psoriasis patients receiving biologics versus those without (all *p* < .05). Pretreatment MALT1 was higher in psoriasis patients who achieved PASI75 (*p* = .001) and PASI90 (*p* < .001) at M6 compared to those who did not achieve that. Subgroup analyses discovered that pretreatment MALT1 had a stronger ability to predict PASI75 and 90 realizations in psoriasis patients receiving biologics (area under the curve [AUC]: 0.723 and 0.808) versus those without (AUC: 0.594 and 0.675).

**Conclusion:**

Blood MALT1 measurement may assist in predicting outcomes in psoriasis patients, especially in those receiving biologics.

## Graphical abstract

Blood mucosa‐associated lymphoid tissue possesses the potential to estimate increased disease activity and favorable treatment response, especially to systemic biologic therapy, in psoriasis patients.

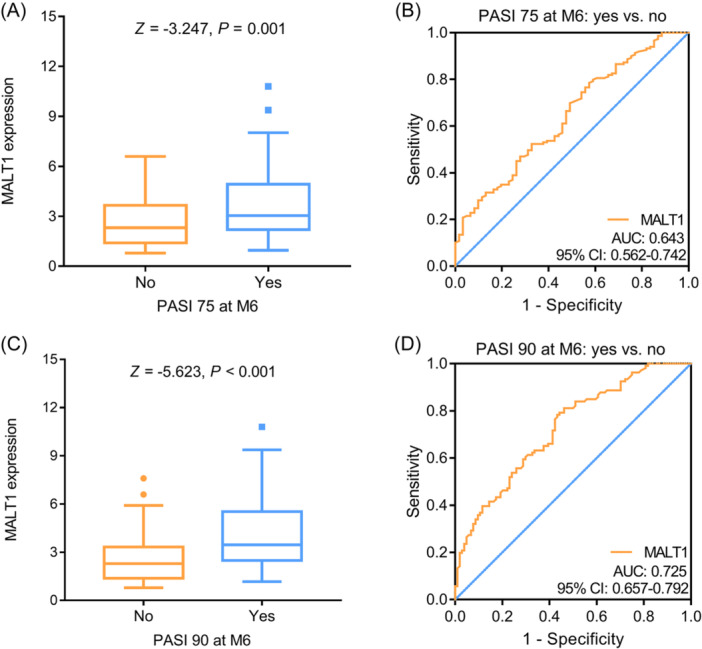

## 1 INTRODUCTION

Psoriasis is a chronic autoimmune skin disease that is caused by abnormal keratinocyte proliferation and commonly manifests as itchy rashes and scaly plaques.^1^ It is reported that the onset of psoriasis has a distinct bimodal age pattern, with the first and second peaks occurring around 30–39 and 60–69 years, respectively.^2^ Generally, treatment options for psoriasis patients include topical therapy, systemic biologic therapy, systemic nonbiologic therapy, and phototherapy.^3^, ^4^, ^5^, ^6^ Unfortunately, psoriasis cannot be completely cured, and a proportion of psoriasis patients do not respond well to the treatments with a psoriasis area severity index (PASI) 90 rate ranging from 10% to 72% within 16 weeks, resulting in decreased quality of life and poor clinical outcomes.^7^, ^8^, ^9^, ^10^, ^11^, ^12^ Therefore, exploring potential markers that estimate treatment response is crucial to improve the management of psoriasis patients.

Mucosa‐associated lymphoid tissue 1 (MALT1) is a special paracaspase, which has been found to mediate inflammatory and immune responses, thereby participating in the pathology of psoriasis.^13^ For instance, one study demonstrates that MALT1 forms a complex with B cell lymphoma/leukemia 10 (BCL10) to amplify the keratinocyte responses to several inflammatory cytokines, then stimulate the lymphocyte‐mediated psoriatic skin inflammation.^14^ Meanwhile, MALT1 regulates T helper (Th) 17 cell and γδ T17 cell differentiation and epidermal hyperplasia to involve in the pathogenesis of psoriasis.^15^ Moreover, MALT1 regulates the caspase recruitment domain protein (CARD)4‐induced production of cytokines and chemokines in keratinocytes, which further causes psoriasis.^16^ Clinically, several studies have reported the role of MALT1 in reflecting treatment response to conventional synthetic disease‐modifying antirheumatic drugs and tumor necrosis factor (TNF) inhibitors in several autoimmune disease patients, including Crohn's disease (CD), rheumatoid arthritis (RA), and ankylosing spondylitis (AS).^17^, ^18^, ^19^, ^20^ However, relevant evidence regarding psoriasis patients is scarce.

The present study aimed to explore the dysregulation of blood MALT1 and its relationship with disease activity and treatment response in psoriasis patients.

## 2 MATERIALS AND METHIDS

### 2.1 Subjects

This study consecutively enrolled 210 psoriasis patients between May 2019 and July 2022. Patients who had the following criteria were eligible for enrollment: (a) clinically diagnosed as psoriasis vulgaris; (b) more than 18‐year‐old; (c) moderate to severe psoriasis with a PASI score ≥8 and the psoriatic area ≥10%); (d) needed to convert a new therapy, or started an initial therapy (for new diagnosis patients); (e) willing to cooperate with follow‐up ≥6 months; and (f) were voluntary cooperation in blood collection for study use. Patients who had any following conditions were excluded: (a) diagnosed as pustular psoriasis or erythrodermic psoriasis; (b) complicated with other autoimmune diseases, other skin diseases, or malignant diseases; and (c) pregnant or lactating women. In addition, 50 disease controls (predominantly atopic dermatitis) and 50 healthy controls (healthy subjects confirmed by physical examinations) were included over the same period, who were included at 35–55 years of age as well as the ratio of 3:2 (male:female) to allow age‐sex matching of psoriasis patients. The study had written informed consent from all subjects and approval from the Ethics Committee with the approval number CDCHLL2023‐427.

### 2.2 Collection

The age and gender of all subjects were collected. Besides, the body mass index (BMI), therapy history, and disease characteristics of psoriasis patients were also recorded. For the blood sample, the peripheral blood of psoriasis patients was collected before conversion to a new treatment or start of initial treatment, as well as peripheral blood of disease controls and healthy controls was collected after enrollment.

### 2.3 Processing

After sample collection, peripheral blood mononuclear cells (PBMCs) were separated, and the MALT1 in PBMCs was detected by reverse transcription‐quantitative polymerase chain reaction (RT‐qPCR). The kits used were as follows: TRIzol™ Reagent (Thermo Fisher Scientific) for total RNA extraction; PrimeScript™ RT reagent Kit (Takar) for reverse transcription; and THUNDERBIRD® SYBR® qPCR Mix (Toyobo) for qPCR. The internal reference was set as GAPDH. The 2^−ΔΔ*C*t^ method was used for calculation. The primers were as follows: MALT1 forward: 5′‐TCTTGGCTGGACAGTTTGTGA‐3′, MALT1 reverse: 5′‐GCTC TCTGGGATGTCGCAA−3′; GAPDH forward: 5′‐GAGTCCACTGGCGTCTTCAC‐3′, GAPDH reverse: 5′‐ATCTTGAGGCTGTTGTCATACTTCT‐3.^18^

### 2.4 Treatment and evaluation

Patients received topical therapy, phototherapy, systemic nonbiologic therapy, or systemic biologics therapy according to the disease status, patient willingness, and the doctor's advice. The data of the current initiating treatment was collected as well. Patients received routine follow‐ups for at least 6 months, during which, 30 patients were lost to follow‐up or quit the study early (18 had poor efficacy, seven were lost to follow‐up, three had adverse reactions, and two quit voluntarily). At the first month (M1), third month (M3), and sixth month (M6), the PASI score was evaluated, and then PASI 75 and PASI 90 were counted. PASI 75 or PASI 90 was calculated as the percentage of patients whose PASI score decreased by 75% or 90% from the time of enrollment.

### 2.5 Statistical analyses

SPSS version 24.0 (IBM Corp.) and GraphPad Prism version 7.0 (GraphPad Prism) were used for analyses and drawings in the study. Normality was analyzed using the Kolmogorov–Smirnov test. Multigroup comparison analysis was completed using the Kruskal–Wallis *H* rank sum test or *χ* ^2^ test; while two‐group comparison analysis was completed using the Wilcoxon rank sum test or *χ* ^2^ test. The post hoc comparison was determined using the Bonferroni test. The distinguishing ability was displayed using the receiver operating characteristic (ROC) curve. Factors related to PASI 75 and PASI 90 at M6 in psoriasis patients were assessed by multivariate logistic regression models with forward‐stepwise method. *p* < .05 was indicated as significant.

## 3 RESULTS

### 3.1 Clinical features of psoriasis patients

Psoriasis patients had a mean age of 47.6 ± 11.0 years and a mean BMI of 28.0 ± 3.6 kg/m^2^. Meanwhile, there were 83 (39.5%) females and 127 (60.5%) males. Regarding therapy history, 206 (98.1%), 200 (95.2%), 166 (79.0%), and 65 (31.0%) patients had a history of topical therapy, phototherapy, systemic nonbiologic therapy, and systemic biologic therapy, respectively. In addition, the disease duration of psoriasis patients was 12.1 ± 6.7 years, and the median (interquartile range [IQR]) psoriatic area was 17.0 (14.0–22.0)%. Moreover, the mean PASI score of psoriasis patients was 13.5 ± 4.8 (Table 1).

**Table 1:** Clinical characteristics of psoriasis patients.

Items	Psoriasis patients (*N* = 210)
Demographic characteristics	
Age (years), mean ± SD	47.6 ± 11.0
Gender, no. (%)	
Female	83 (39.5)
Male	127 (60.5)
BMI (kg/m^2^), mean ± SD	28.0 ± 3.6
Therapy history	
History of topical therapy, no. (%)	
No	4 (1.9)
Yes	206 (98.1)
History of phototherapy, no. (%)	
No	10 (4.8)
Yes	200 (95.2)
History of systemic nonbiologic therapy, no. (%)	
No	44 (21.0)
Yes	166 (79.0)
History of systemic biologic therapy, no. (%)	
No	145 (69.0)
Yes	65 (31.0)
Disease characteristics	
Disease duration (years), mean ± SD	12.1 ± 6.7
Psoriatic area (%), median (IQR)	17.0 (14.0–22.0)
PASI score, mean ± SD	13.5 ± 4.8

Abbreviations: BMI, body mass index; IQR, interquartile range; PASI, psoriasis area severity index; SD, standard deviation.

### 3.2 Comparison of blood MALT1 among psoriasis patients, disease controls, and healthy controls

**Figure 1:**
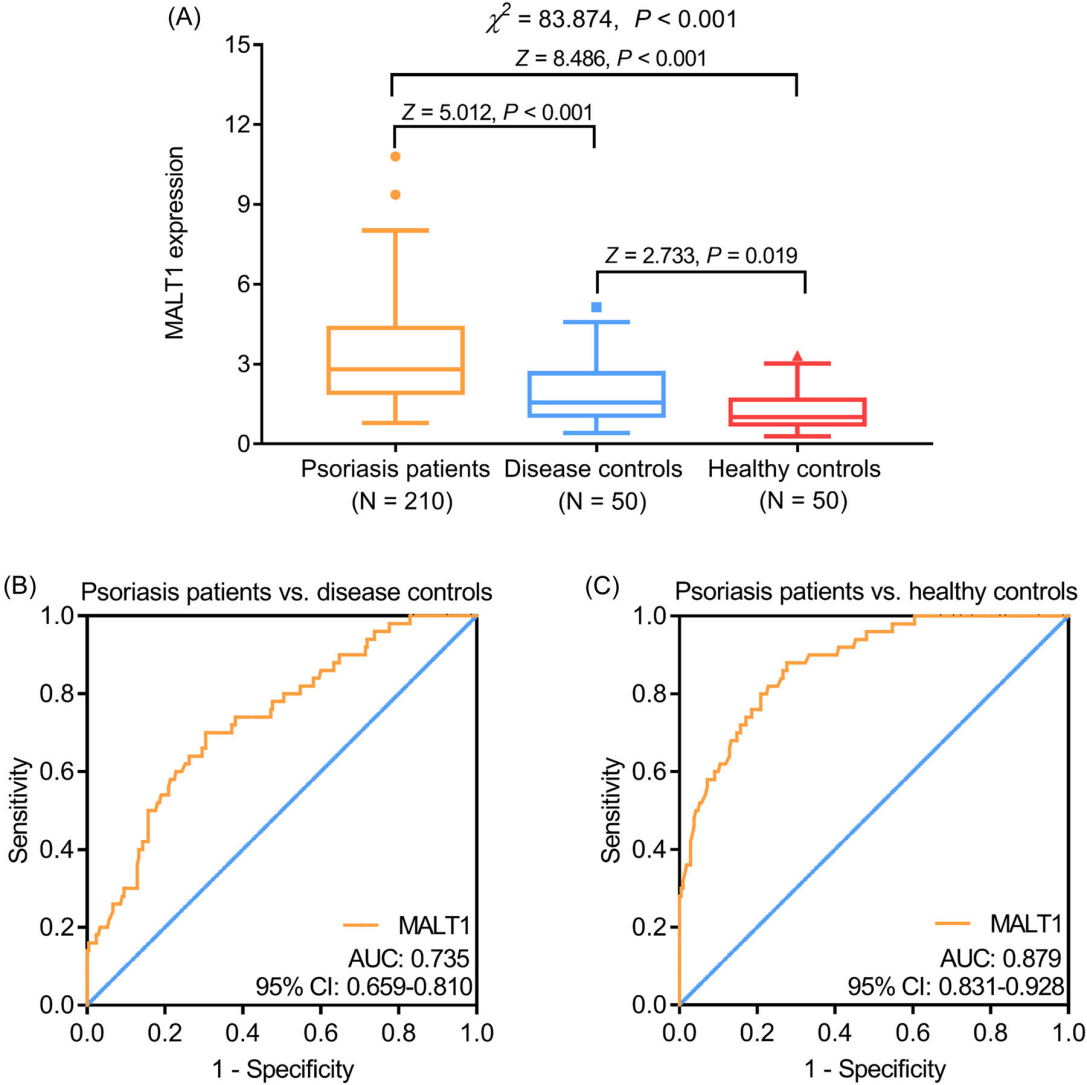
Blood MALT1 in psoriasis patients, disease controls, and healthy controls. Comparison of blood MALT1 among psoriasis patients, disease controls, and healthy controls (A); the discriminative ability of blood MALT1 between psoriasis patients and disease controls (B), as well as between psoriasis patients and healthy controls (C). MALT1, mucosa‐associated lymphoid tissue 1.

Blood MALT1 was the highest in psoriasis patients, followed by disease controls, and the lowest in healthy controls (*p* < .001). The post hoc comparison revealed that blood MALT1 was increased in psoriasis patients compared to disease controls and healthy controls (both *p* < .001); meanwhile, blood MALT1 was also elevated in disease controls versus healthy controls (*p* = .019) (Figure 1A). The ROC curve suggested that blood MALT1 had an acceptable ability to discriminate psoriasis patients from disease controls with an area under the curve (AUC) (95% confidence interval [CI]) of 0.735 (0.659–0.810) (Figure 1B). Notably, blood MALT1 had a good ability to discriminate psoriasis patients from healthy controls with an AUC (95% CI) of 0.879 (0.831–0.928) (Figure 1C).

### 3.3 Correlation of blood MALT1 with current initiating treatment in psoriasis patients

Notably, 154 (73.3%), 131 (62.4%), 157 (47.8%), and 79 (37.6%) patients had a current initiating topical therapy, phototherapy, systemic nonbiologic therapy, and systemic biologic therapy, respectively. Regarding systemic nonbiologic therapy, 75 (47.8%) patients received acitretin, 55 (35.0%) patients received methotrexate (MTX), and 27 (17.2%) patients received cyclosporin A (CyA). With respect to systemic biologic therapy, 28 (35.4%) patients received adalimumab, 21 (26.6%) patients received ustekinumab, 17 (21.5%) patients received etanercept, seven (8.9%) patients received infliximab, and six (7.6%) patients received secukinumab (Table 2).

**Table 2:** Current initiating treatment of psoriasis patients.

Items	Psoriasis patients (*N* = 210)
Topical therapy, no. (%)	
No	56 (26.7)
Yes	154 (73.3)
Phototherapy, no. (%)	
No	79 (37.6)
Yes	131 (62.4)
Systemic nonbiologic therapy, no. (%)	
No	53 (25.2)
Yes	157 (47.8)
Regimens of systemic nonbiologic therapy, no. (%)	
Acitretin	75 (47.8)
MTX	55 (35.0)
CyA	27 (17.2)
Systemic biologic therapy, no. (%)	
No	131 (62.4)
Yes	79 (37.6)
Regimens of systemic biologic therapy, no. (%)	
Adalimumab	28 (35.4)
Ustekinumab	21 (26.6)
Etanercept	17 (21.5)
Infliximab	7 (8.9)
Secukinumab	6 (7.6)

Abbreviations: CyA, cyclosporin A; MTX, methotrexate.

**Figure 2:**
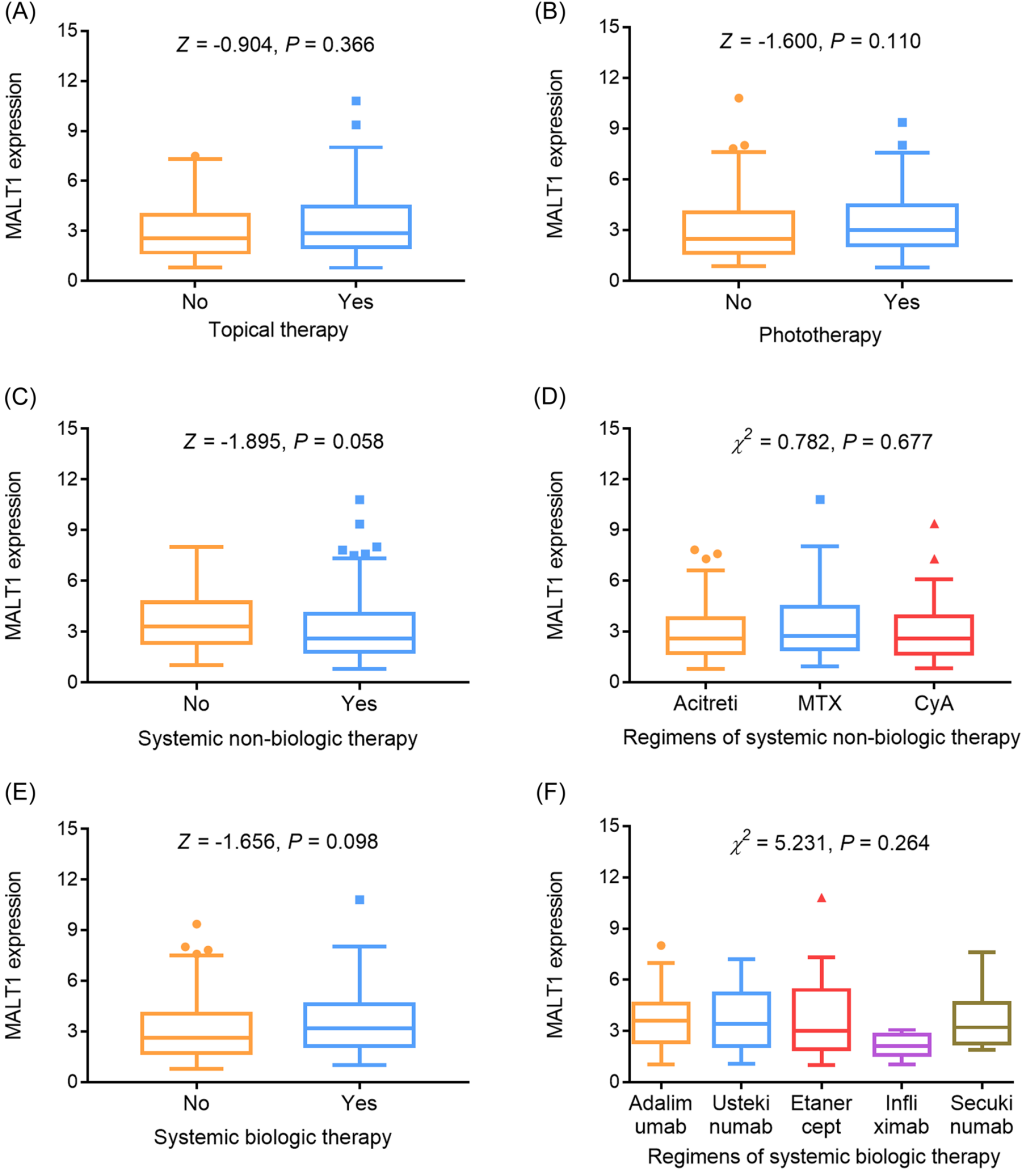
Relationship of blood MALT1 with current initiating treatment in psoriasis patients. Correlation of blood MALT1 with topical therapy (A), phototherapy (B), systemic nonbiologic therapy (C), regimens of systemic nonbiologic therapy (D), systemic biologic therapy (E), and regimens of systemic biologic therapy (F) in psoriasis patients. MALT1, mucosa‐associated lymphoid tissue 1.

Blood MALT1 was not correlated with topical therapy (*p* = .366) (Figure 2A) or phototherapy (*p* = .110) (Figure 2B). Meanwhile, blood MALT1 was also not related to systemic nonbiologic therapy (*p* = .058) (Figure 2C) or specific regimens of systemic nonbiologic therapy (*p* = .677) (Figure 2D). Additionally, blood MALT1 was not associated with systemic biologic therapy (*p* = .098) (Figure 2E) or specific regimens of systemic biologic therapy either (*p* = .264) (Figure 2F).

### 3.4 Correlation of blood MALT1 with clinical features in psoriasis patients

Blood MALT1 was positively related to BMI (*r* = .162, *p* = .019) and PASI score (*r* = .260, *p* < .001) in psoriasis patients. Whereas blood MALT1 was not correlated with other clinical characteristics (all *p* > .05). Notably, blood MALT1 was also not associated with treatment history, including history of topical therapy (*p* = .404), history of phototherapy (*p* = .334), history of systemic nonbiologic therapy (*p* = .588), and history of systemic biologic therapy (*p* = .265) (Table 3).

**Table 3:** Correlation of MALT1 expression with clinical characteristics in psoriasis patients.

Items	MALT1 expression, median (IQR)	*Z/r* Value	*p* Value
Demographic characteristics			
Age (years)^a^	—	−.084	.228
Gender^b^		−.453	.651
Female	2.530 (1.660–4.860)		
Male	2.880 (2.020–4.060)		
BMI (kg/m^2^)^a^	—	.162	.019
Therapy history			
History of topical therapy^b^		−.835	.404
No	2.440 (1.600–3.168)		
Yes	2.800 (1.935–4.383)		
History of phototherapy^b^		−.965	.334
No	2.135 (1.638–3.635)		
Yes	2.830 (1.948–4.360)		
History of systemic nonbiologic therapy^b^		−.541	.588
No	3.035 (2.288–4.723)		
Yes	2.770 (1.880–4.360)		
History of systemic biologic therapy^b^		−1.114	.265
No	2.900 (1.900–4.575)		
Yes	2.750 (1.930–3.670)		
Disease characteristics			
Disease duration (years)^a^	—	.083	.233
Psoriatic area (%)^a^	—	.130	.059
PASI score^a^	—	.260	<.001

Abbreviations: BMI, body mass index; IQR, interquartile range; MALT1, mucosa‐associated lymphoid tissue 1; PASI, psoriasis area severity index.

^a^ Analyzed using Spearman's rank correlation test, the data was displayed by *r* value and *p* value.

^b^ Analyzed using Mann–Whitney *U* test, the data was displayed by median (IQR), *Z* value, and *p* value.

### 3.5 PASI 75 and 90 rates in psoriasis patients

**Figure 3:**
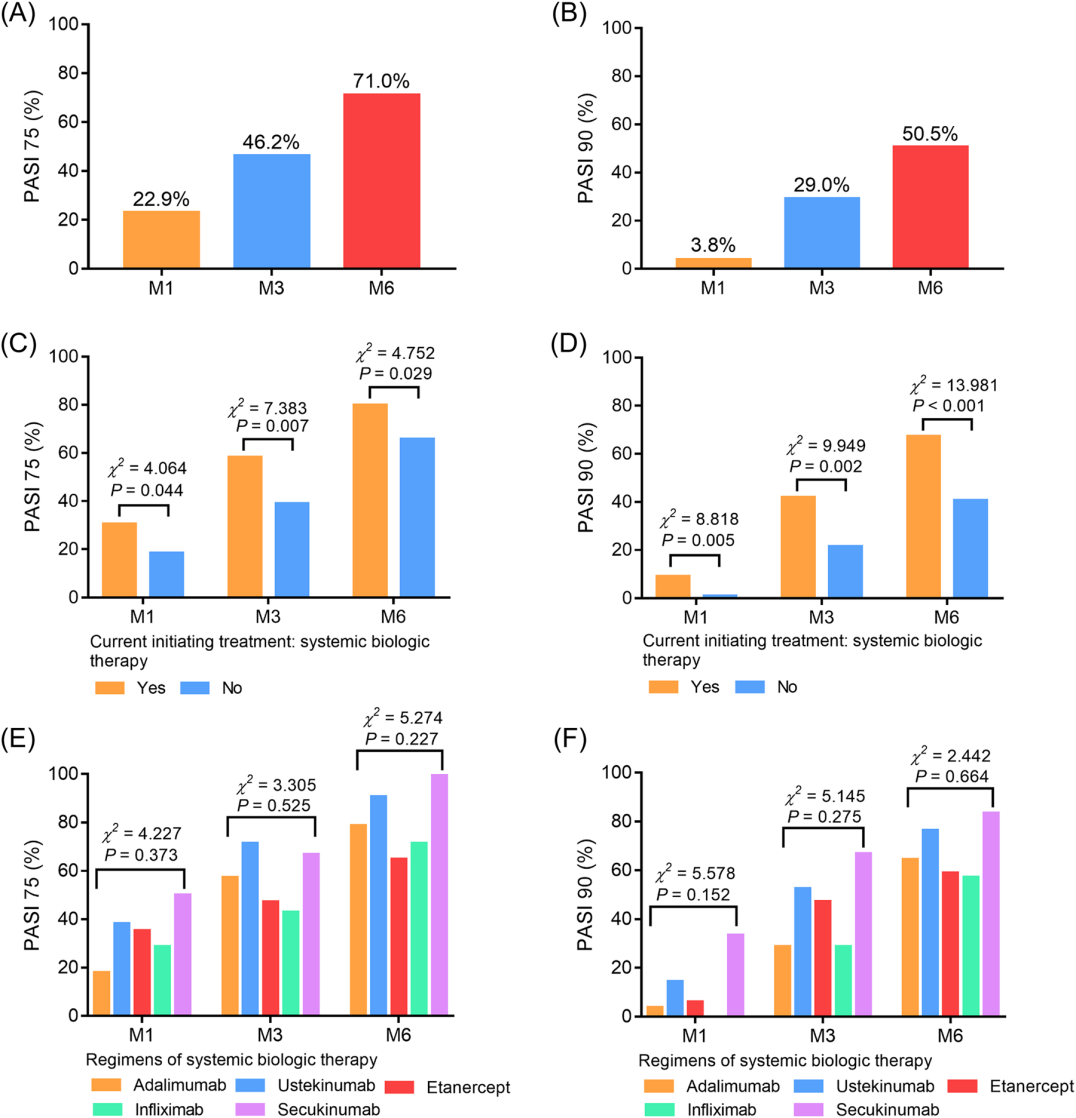
Treatment response rates in psoriasis patients. PASI 75 (A) and 90 (B) rates at M1, M3, and M6 in psoriasis patients; comparison of PASI 75 (C) and 90 (D) rates at M1, M3, and M6 between patients with current initiating systemic biologic treatment and those without that; comparison of PASI 75 (E) and 90 (F) rates at M1, M3, and M6 among patients who received adalimumab, ustekinumab, etanercept, infliximab, and secukinumab. PASI, psoriasis area severity index.

Notably, 22.9%, 46.2%, and 71.0% of psoriasis patients achieved PASI 75 at M1, M3, and M6, respectively (Figure 3A). Meanwhile, 3.8%, 29.0%, and 50.5% of psoriasis patients achieved PASI 90 at M1, M3, and M6, respectively (Figure 3B). Notably, the current initiating systemic biologic therapy was related to a higher PASI 75 rate at M1 (*p* = .044), M3 (*p* = .007), and M6 (*p* = .029) (Figure 3C). Meanwhile, the current initiating systemic biologic therapy was also correlated with a higher PASI 90 rate at M1 (*p* = .005), M3 (*p* = .002), and M6 (*p* < .001) (Figure 3D). However, specific regimens of systemic biologic therapy were not related to PASI 75 (Figure 3E) and 90 (Figure 3F) rates at M1, M3, and M6 (all *p* > .05).

### 3.6 Correlation of blood MALT1 with PSAI 75 and 90 at M6 in psoriasis patients

**Figure 4:**
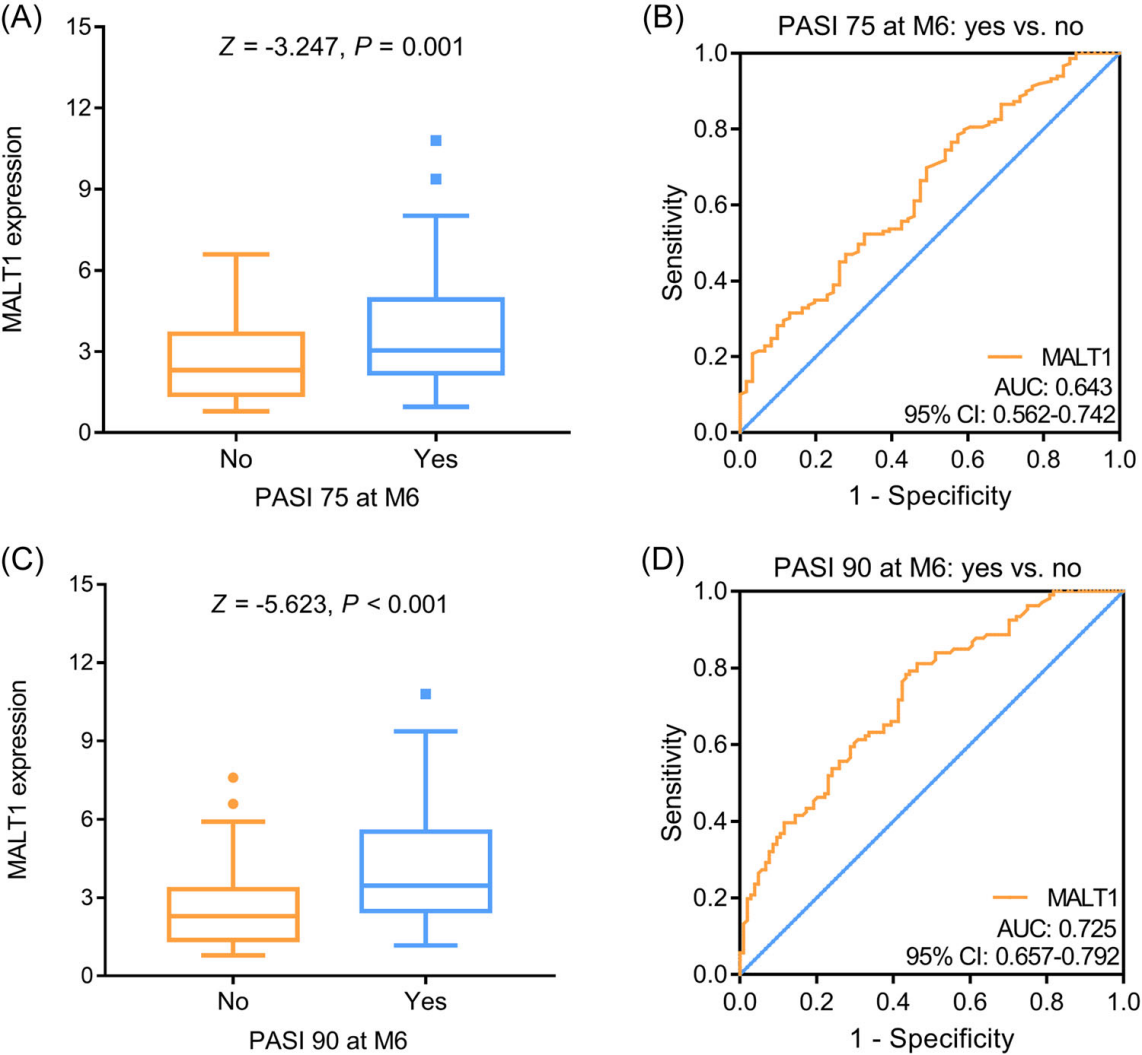
Relationship of blood MALT1 with treatment response in psoriasis patients. Comparison (A) and discriminative ability (B) of blood MALT1 between psoriasis patients who achieved PASI 75 at M6 and those who did not achieve that; comparison (C) and discriminative ability (D) of blood MALT1 between psoriasis patients who achieved PASI 90 at M6 and those who did not achieve that. MALT1, mucosa‐associated lymphoid tissue 1; PASI, psoriasis area severity index.

Blood MALT1 was increased in psoriasis patients who achieved PASI 75 at M6 compared to those who did not achieve that (*p* = .001) (Figure 4A). The ROC curve suggested that blood MALT1 only had a weak capability to discriminate patients who achieved PASI 75 at M6 from those who did not achieve that (AUC [95% CI]: 0.643 [0.562–0.742]) (Figure 4B). In addition, blood MALT1 was also elevated in psoriasis patients who achieved PASI 90 at M6 versus those who did not achieve that (*p* < .001) (Figure 4C). The ROC curve revealed that blood MALT1 had an acceptable ability to distinguish patients who achieved PASI 90 at M6 from those who did not achieve that (AUC [95% CI]: 0.725 [0.657–0.792]) (Figure 4D). Multivariate logistic regression analysis suggested that blood MALT1 was independently related to achieving PASI 75 (odds ratio = 1.362, *p* = .002) and PASI 90 (odds ratio = 1.629, *p* < .001) at M6 in psoriasis patients (Table S1).

**Figure 5:**
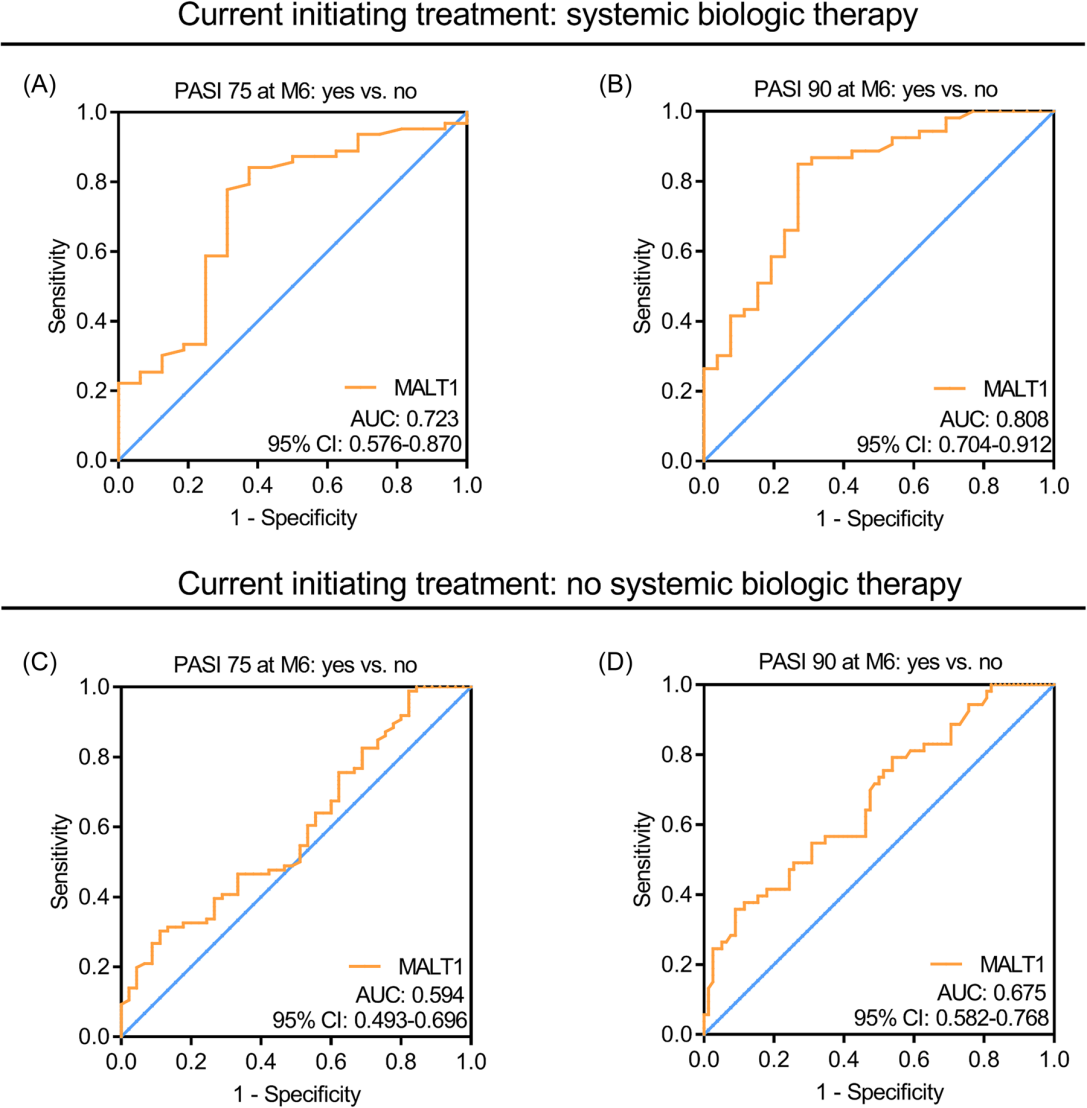
Subgroup analyses based on current initiating systemic biologic therapy in psoriasis patients. Blood MALT1 for discriminating psoriasis patients with current initiating systemic biological therapy who achieved PASI 75 (A) and 90 (B) at M6 from those who did not achieve that; blood MALT1 for discriminating psoriasis patients without current initiating systemic biological therapy who achieved PASI 75 (C) and 90 (D) at M6 from those who did not achieve that. MALT1, mucosa‐associated lymphoid tissue 1; PASI, psoriasis area severity index.

Further subgroup analyses found that in patients with current initiating systemic biologic therapy, blood MALT1 had an acceptable ability to discriminate patients who achieved PASI 75 (AUC [95% CI]: 0.723 [0.576–0.870]) (Figure 5A) and 90 (AUC [95% CI]: 0.808 [0.704–0.912]) (Figure 5B) at M6 from those who did not achieve that. While in patients without current initiating systemic biologic therapy, blood MALT1 could barely discriminate patients who achieved PASI 75 (AUC [95% CI]: 0.594 [0.493–0.696]) (Figure 5C) and 90 (AUC [95% CI]: 0.675 [0.582–0.768]) (Figure 5D) at M6 from those who did not achieve that.

## 4 DISCUSSION

MALT1, as a scaffold protein, plays a fundamental role in immunity and inflammation, which is responsible for the occurrence of various autoimmune diseases.^21^ Recently, several clinical studies have displayed an increase in MALT1 in autoimmune disease patients.^17^, ^22^, ^23^ However, rare studies report the dysregulation of MALT1 in psoriasis patients. In accordance with these previous studies,^17^, ^22^, ^23^ the present study also figured out that blood MALT1 was increased in psoriasis patients compared with disease controls and healthy controls. The potential reasons could be that (1) MALT1 might regulate the c‐Jun pathway to facilitate the hyperproliferation of keratinocytes and the production of chemokines to induce psoriasis.^15^ (2) MALT1 might also form a complex with BCL10 to initiate psoriatic skin inflammation.^14^ As a result, increased blood MALT1 reflected a higher psoriasis risk.

The relationship of MALT1 with clinical features in several autoimmune diseases has been revealed by previous studies.^17^, ^18^, ^20^ According to a study, MALT1 is positively related to C‐reactive protein (CRP), erythrocyte sedimentation rate (ESR), and clinical disease activity index (CDAI) in CD patients.^17^ In addition, another study discovers that MALT1 reflects increased disease activity of RA with a positive correlation with CRP, ESR, and Disease Activity Score‐28 (DAS28) in RA patients.^18^ In this study, it was found that blood MALT1 was positively correlated with BMI and PASI score in psoriasis patients. Regarding BMI, it could be explained by that MALT1 might form a complex with BCL10 and CARD3 to regulate insulin resistance, leading to an increase in BMI.^24^, ^25^ With respect to the PASI score, MALT1 could facilitate Th17 cell and γδ T17 cell differentiation, inflammatory cytokine (such as TNF‐α, interleukin [IL]−17A, and IL‐1β) production, and keratinocyte proliferation to eventually lead to psoriasis symptoms, including hyperkeratosis of the epidermal tissue, loss of the granular layer and epidermal microabscesses, contributing to a higher PASI score.^14^, ^15^, ^16^

Systemic biologic therapy has recently received a lot of attention due to its rapid onset of action, safety, and ability to target specific immune system‐mediated inflammatory cytokines to attenuate psoriasis activity.^3^ In this study, several interesting findings regarding systemic biologic therapy in psoriasis patients were disclosed. Firstly, it was found that PASI 75 and 90 rates at M1, M3, and M6 were increased in psoriasis patients with current initiating systemic biologic therapy versus those without that. A potential explanation would be that biological drugs could target TNF‐α, IL‐17A, and IL‐23 to inhibit inflammation, thereby alleviating epidermal proliferation, thickening, and massive desquamation.^26^, ^27^ Hence, PASI 75 and 90 rates were higher in patients with systemic biologic therapy. Secondly, this study discovered that blood MALT1 was elevated in psoriasis patients who achieved PASI 75 and 90 at M6 versus those who did not achieve that. A reason behind this might be that MALT1 could regulate immune response, inflammation, and keratinocyte proliferation to facilitate the progression of psoriasis; meanwhile, patients with exacerbated psoriasis might benefit more from treatments.^14^, ^15^, ^16^ Therefore, elevated blood MALT1 was related to achieving PASI 75 and 90 at M6 in psoriasis patients. Thirdly, from the results of subgroup analyses, blood MALT1 seemed to have a stronger ability to distinguish psoriasis patients with current initiating systemic biologic therapy who achieved PASI 75 and 90 at M6 from those who did not achieve that. This finding might indicate that blood MALT1 might predominantly predict treatment response to systemic biologic therapy, while its ability to predict treatment response to other therapies was relatively weak in psoriasis patients, and the predictive ability of blood MALT1 for whole treatment response was mainly derived from the former.

The findings of this study might provide a reference that the early detection of blood MALT1 was meaningful to predict treatment response in psoriasis patients. However, some limitations still existed. (1) The longitudinal change of blood MALT1 was not investigated in this study; however, this would be meaningful to monitor the disease progression of psoriasis patients. (2) This study figured out that blood MALT1 had the potential to predict treatment response to systemic biologic therapy in psoriasis patients, which indicated that blood MALT1 participated in the process of systemic biologic therapy in attenuating psoriasis activity; however, this speculation was warranted to validate. (3) Considering the long‐term treatment responses of a proportion of psoriasis patients were unsatisfactory enough, it was meaningful to prolong the follow‐up duration to explore the ability of blood MALT1 for predicting long‐term treatment response in psoriasis patients. (4) The sample size of this study was inadequate; therefore, the findings of this study might be hard to generalize.

In summary, blood MALT1 may possess the potential to estimate increased disease activity and favorable treatment response, especially to systemic biologic therapy, in psoriasis patients. Considering psoriasis patients require long‐term treatments, early detection of blood MALT1 before the initiation of treatments, especially systemic biologic therapy, may be helpful in predicting the therapeutic benefits in these patients. However, restricted by the sample size of this study, the number of patients receiving systemic biologic therapy is much smaller. Therefore, the generalization of this study should be further verified.

## AUTHOR CONTRIBUTIONS

**Qiaoli Liu**: Conceptualization (equal); methodology (equal); writing—original draft (lead); formal analysis (lead); writing—review and editing (equal). **Yanfeng Zhang**: Conceptualization (equal); methodology (equal); writing—original draft (lead); writing—review and editing (equal). **Bing Xu**: Writing—original draft (equal); formal analysis (equal); writing—review and editing (equal). **Xiaobo Jin**: Writing—original draft (equal); writing—review and editing (equal). **Tao Yang**: Writing—original draft (equal); writing—review and editing (equal). **Leiqiang Fan**: conceptualization (lead); formal analysis (equal); writing—review and editing (equal).

## CONFLICT OF INTEREST STATEMENT

The authors declare no conflict of interest.

## ETHICS STATEMENT

The study had written informed consent from all subjects and approval from the Ethics Committee.

## Supporting information

Supporting information.

## Data Availability

The data sets used and/or analyzed during the current study are available from the corresponding author on reasonable request.

## References

[1] Griffiths CEM , Armstrong AW , Gudjonsson JE , Barker JNWN . Psoriasis. Lancet. 2021;397(10281):1301‐1315. 33812489 10.1016/S0140-6736(20)32549-6

[2] Iskandar IYK , Parisi R , Griffiths CEM , Ashcroft DM . Systematic review examining changes over time and variation in the incidence and prevalence of psoriasis by age and gender. Br J Dermatol. 2021;184(2):243‐258. 32358790 10.1111/bjd.19169

[3] Armstrong AW , Read C . Pathophysiology, clinical presentation, and treatment of psoriasis: a review. JAMA. 2020;323(19):1945‐1960. 32427307 10.1001/jama.2020.4006

[4] Elmets CA , Korman NJ , Prater EF , et al. Joint AAD‐NPF guidelines of care for the management and treatment of psoriasis with topical therapy and alternative medicine modalities for psoriasis severity measures. J Am Acad Dermatol. 2021;84(2):432‐470. 32738429 10.1016/j.jaad.2020.07.087

[5] Menter A , Gelfand JM , Connor C , et al. Joint American Academy of Dermatology‐National Psoriasis Foundation guidelines of care for the management of psoriasis with systemic nonbiologic therapies. J Am Acad Dermatol. 2020;82(6):1445‐1486. 32119894 10.1016/j.jaad.2020.02.044

[6] Korman NJ . Management of psoriasis as a systemic disease: what is the evidence? Br J Dermatol. 2020;182(4):840‐848. 31225638 10.1111/bjd.18245 PMC7187293

[7] Lebwohl M , Thaçi D , Warren RB . Addressing challenges associated with long‐term topical treatment and benefits of proactive management in patients with psoriasis. J Eur Acad Dermatol Venereol. 2021;35(suppl 1):35‐41. 33619776 10.1111/jdv.17053 PMC7985873

[8] Puig L , Costanzo A , Muñoz‐Elías EJ , et al. The biological basis of disease recurrence in psoriasis: a historical perspective and current models. Br J Dermatol. 2022;186(5):773‐781. 34939663 10.1111/bjd.20963 PMC9374062

[9] Masson Regnault M , Shourick J , Jendoubi F , Tauber M , Paul C . Time to relapse after discontinuing systemic treatment for psoriasis: a systematic review. Am J Clin Dermatol. 2022;23(4):433‐447. 35489008 10.1007/s40257-022-00679-y PMC9055370

[10] Ujiie H , Rosmarin D , Schön MP , et al. Unmet medical needs in chronic, non‐communicable inflammatory skin diseases. Front Med. 2022;9:875492. 10.3389/fmed.2022.875492 PMC9218547 35755063

[11] Armstrong AW , Puig L , Joshi A , et al. Comparison of biologics and oral treatments for plaque psoriasis: a meta‐analysis. JAMA Dermatol. 2020;156(3):258‐269. 32022825 10.1001/jamadermatol.2019.4029 PMC7042876

[12] Armstrong AW , Soliman AM , Betts KA , et al. Comparative efficacy and relative ranking of biologics and oral therapies for moderate‐to‐severe plaque psoriasis: a network meta‐analysis. Dermatol Ther. 2021;11(3):885‐905. 10.1007/s13555-021-00511-1 PMC8163943 33788177

[13] Hailfinger S , Schulze‐Osthoff K . The paracaspase MALT1 in psoriasis. Biol Chem. 2021;402(12):1583‐1589. 34192836 10.1515/hsz-2021-0250

[14] Kurgyis Z , Vornholz L , Pechloff K , et al. Keratinocyte‐intrinsic BCL10/MALT1 activity initiates and amplifies psoriasiform skin inflammation. Sci Immunol. 2021;6(65):eabi4425. 34826258 10.1126/sciimmunol.abi4425

[15] Xia X , Cao G , Sun G , et al. GLS1‐mediated glutaminolysis unbridled by MALT1 protease promotes psoriasis pathogenesis. J Clin Invest. 2020;130(10):5180‐5196. 32831293 10.1172/JCI129269 PMC7524468

[16] Afonina IS , Van Nuffel E , Baudelet G , et al. The paracaspase MALT1 mediates CARD14‐induced signaling in keratinocytes. EMBO Rep. 2016;17(6):914‐927. 27113748 10.15252/embr.201642109 PMC5278603

[17] Dong X , Chen X , Ren Y . MALT1 reflects inflammatory cytokines, disease activity, and its chronological change could estimate treatment response to infliximab in Crohn's disease patients. J Clin Lab Anal. 2022;36(10):e24650. 36036788 10.1002/jcla.24650 PMC9550982

[18] Wang Q , Wang Y , Liu Q , et al. MALT1 regulates Th2 and Th17 differentiation via NF‐κB and JNK pathways, as well as correlates with disease activity and treatment outcome in rheumatoid arthritis. Front Immunol. 2022;13:913830. 35967391 10.3389/fimmu.2022.913830 PMC9367691

[19] Ye Z , Chen L , Fang Y , Zhao L . Blood MALT1, Th1, and Th17 cells are dysregulated, inter‐correlated, and correlated with disease activity in rheumatoid arthritis patients; meanwhile, MALT1 decline during therapy relates to treatment outcome. J Clin Lab Anal. 2022;36(1):e24112. 34788483 10.1002/jcla.24112 PMC8761436

[20] Yuan J , Xiang L , Wang F , et al. MALT1 positively relates to Th17 cells, inflammation/activity degree, and its decrement along with treatment reflects TNF inhibitor response in ankylosing spondylitis patients. J Clin Lab Anal. 2022;36(7):e24472. 35622982 10.1002/jcla.24472 PMC9279967

[21] Demeyer A , Staal J , Beyaert R . Targeting MALT1 proteolytic activity in immunity, inflammation and disease: good or bad? Trends Mol Med. 2016;22(2):135‐150. 26787500 10.1016/j.molmed.2015.12.004

[22] Wang F , Liu G , Xiang L , et al. Mucosa‐associated lymphoid tissue lymphoma translocation protein 1 in rheumatoid arthritis: longitudinal change after treatment and correlation with treatment efficacy of tumor necrosis factor inhibitors. J Clin Lab Anal. 2022;36(6):e24449. 35500150 10.1002/jcla.24449 PMC9169166

[23] Wang M , Huang L , Peng L , et al. MALT1 serves as a biomarker for estimating disease risk of lupus nephritis: a prospective case‐control study. Ann Transl Med. 2022;10(16):874. 36111027 10.21037/atm-22-3442 PMC9469135

[24] Gui Z , Zhang Y , Zhang A , Xia W , Jia Z . CARMA3: a potential therapeutic target in non‐cancer diseases. Front Immunol. 2022;13:1057980. 36618379 10.3389/fimmu.2022.1057980 PMC9815110

[25] Wu H , Ballantyne CM . Metabolic inflammation and insulin resistance in obesity. Circ Res. 2020;126(11):1549‐1564. 32437299 10.1161/CIRCRESAHA.119.315896 PMC7250139

[26] Singh R , Koppu S , Perche PO , Feldman SR . The cytokine mediated molecular pathophysiology of psoriasis and its clinical implications. Int J Mol Sci. 2021;22(23):12793. 34884596 10.3390/ijms222312793 PMC8657643

[27] Balato A , Scala E , Balato N , et al. Biologics that inhibit the Th17 pathway and related cytokines to treat inflammatory disorders. Expert Opin Biol Ther. 2017;17(11):1363‐1374. 28791896 10.1080/14712598.2017.1363884

