# Supplementary material for: Blood MALT1 expression could help predict treatment outcomes in psoriasis patients, especially in those receiving biologics
Source: Immun Inflamm Dis. 2024 Apr 5;12(4):e1235. doi: 10.1002/iid3.1235 (PMC10996377; doi:10.1002/iid3.1235)
Supplement: Supplementary file 1 — Supporting information. [file IID3-12-e1235-s001.docx]

**Supplementary Table 1.** Multivariate logistic regression models for PASI 75 and PASI 90 at M6 in psoriasis patients.

| Items | *P* value | OR (95% CI) |
| --- | --- | --- |
| **For PASI 75 at M6** |  |  |
| MALT1 expression | 0.002 | 1.362 (1.118-1.660) |
| Current initiating treatment of non-systemic biologic therapy | 0.010 | 0.315 (0.132-0.756) |
| **For PASI 90 at M6** |  |  |
| MALT1 expression | <0.001 | 1.629 (1.345-1.974) |
| Current initiating treatment of systemic biologic therapy | 0.001 | 2.969 (1.584-5.567) |

PASI, psoriasis area severity index; M6, 6^th^ month; OR, odds ratio; CI, confidence interval; MALT1, mucosa-associated lymphoid tissue 1.
